# MedHopper: an agentic RAG-LLM system for multi-hop biomedical QA

**DOI:** 10.1093/database/baag047

**Published:** 2026-08-13

**Authors:** Rustam Ruslanovich Taktashov, Nadezhda Yurievna Biziukova, Alexander Viktorovich Dmitriev, Olga Aleksandrovna Tarasova

**Affiliations:** Institute of Biomedical Chemistry, 10 bld. 8, Pogodinskaya str., 119121 Moscow, Russia; Institute of Biomedical Chemistry, 10 bld. 8, Pogodinskaya str., 119121 Moscow, Russia; Institute of Biomedical Chemistry, 10 bld. 8, Pogodinskaya str., 119121 Moscow, Russia; Institute of Biomedical Chemistry, 10 bld. 8, Pogodinskaya str., 119121 Moscow, Russia

## Abstract

This multi-hop biomedical QA system combines dense retrieval with a bounded agentic workflow for the MedHopQA (BioCreative IX) task, which requires short, entity-centric answers from a Wikipedia-derived corpus evaluated via exact match with normalization. Our approach replaces prior prompt decomposition with a controlled state machine that routes questions into four strategies (direct, definition, intersection, and multi-hop). The pipeline performs dense retrieval with cross-encoder reranking, executes up to three hops of intermediate entity extraction when needed, and applies answer validation with a bounded query-repair loop. On the MedHopQA test set (*N* = 1000), MedHopper achieves 0.55 Exact Match (MedHopQA evaluation metric) under official CodaBench evaluation. Ablation confirms capped multi-hop execution, reranking, and query repair as primary contributors; validation modules provide smaller consistent gains. Analysis reveals persistent challenges under exact-match scoring, including answer-type inconsistency (chromosome vs cytoband; yes/no vs symptom) and surface variation across plausible lexicalizations. All experiments were run on a single consumer GPU (RTX 5080) with no API dependencies, producing deterministic outputs at zero marginal cost per query.

**Database URL:** https://github.com/RustT883/MedHopper.

## Introduction

Large language models (LLMs) are increasingly used for biomedical natural language processing (NLP) tasks, including question answering, summarization, and interactive assistance. Reliability remains a major obstacle because generative models can produce fluent but unsupported or incorrect text. Hallucination surveys document ungrounded generation and stress the need for grounding and verification in high-reliability contexts [1]. Clinical evaluation work further argues that medical use requires structured assessment protocols and error taxonomies rather than coarse accuracy metrics alone [2].

Deploying LLMs in biomedical research often faces practical barriers: cloud APIs incur recurring costs, raise data confidentiality concerns, and provide non-deterministic outputs that hinder reproducibility. Local deployment on commodity hardware avoids these issues, but requires efficient, low-resource architectures. This work focuses on building a fully local, transparent multi-hop question answering (QA) system that runs on a single consumer GPU, without sacrificing structured reasoning or answer grounding.

Retrieval-augmented generation (RAG) reduces unsupported generation by conditioning the LLM on retrieved documents [3]. In open-domain or corpus-grounded QA, retrieval quality often dominates correctness because the generator cannot infer facts not retrieved. This is more pronounced in multi-hop QA, where a single query derived from the original question may lack the intermediate bridge entity needed to retrieve the second evidence piece. Multi-hop QA benchmarks over Wikipedia were designed to capture this failure mode and motivate explicit multi-step evidence discovery [4, 5].

### LLMs and RAG in biomedicine

Biomedical QA has long been developed in shared task settings with expert-authored questions, gold answers, and evidence-oriented evaluation. BioASQ is a central example, establishing evaluation sensitive to biomedical terminology and concept variation [6, 7]. The BioASQ-QA corpus reinforces that evaluation must handle synonym recognition and normalization [8]. Surveys classify biomedical QA systems into information retrieval (IR)-based QA, machine reading comprehension, knowledge-based QA, and hybrid pipelines, highlighting long-tail entities, abbreviation ambiguity, and the need for provenance [9].

With LLM-based QA, these issues interact with generative model reliability. Hallucination surveys [1] and clinical evaluation frameworks [2] motivate external grounding and verification. Domain-adapted encoders improved biomedical representations: BioBERT [10], SciBERT [11], and clinical LLMs like Med42-v2 [12]. Even with domain adaptation, short-answer biomedical QA often requires explicit grounding and constraints to output specific entity strings rather than generic labels, especially under strict scoring [13].

RAG conditions generation on retrieved evidence to improve factuality and handle knowledge beyond model parameters [3]. Aligning the retrieval corpus to the benchmark provenance is a design choice. MedHopQA questions are curated from Wikipedia pages, motivating Wikipedia-based corpora for retrieval-centric systems [13, 14].

Dense retrieval uses learned embeddings and similarity search. Sentence-BERT [15], ColBERT [16], BEIR [17], and MS MARCO [18] form the general retrieval toolbox. In biomedical IR, MedCPT provides a contrastively trained retriever–reranker and is used for MedHopQA’s concept-level scoring [13, 19].

Hybrid retrieval with reciprocal rank fusion (RRF) can increase recall [20]. Our initial MedHopQA system used hybrid retrieval and RRF [14]. The current approach uses dense retrieval only. Two-stage retrieval (large candidate set then reranking) is common; FlashRank is a lightweight reranker used in RAG pipelines [21].

Fine-tuning improves instruction adherence but does not replace retrieval when answers depend on external evidence. MedHopper is retrieval-centric, combining retrieval, reranking, and output constraints [3]. MedHopQA evaluates short, entity-like answers with Exact Match (EM) and concept-level scoring, motivating explicit canonicalization and type control [13].

### LLM-based agents; ReAct and agentic workflow

Multi-hop QA benchmarks formalize questions that require integrating evidence across multiple documents. HotpotQA introduced Wikipedia-based multi-hop questions with supporting fact supervision [4]. 2WikiMultiHopQA provides evidence annotations [5]. MuSiQue constructs questions by composing single-hop questions [22]. For retrieval-based QA systems, a one-shot query may miss the required evidence chain, because the text needed for the second passage may become explicit only after recovering an intermediate entity [4, 5, 23]. Thus, many multi-hop pipelines treat retrieval iteratively: retrieve, extract a bridge entity, then issue refined queries. End-to-end beam retrieval constructs passage chains rather than independent sets [24]. MedHopper implements this via hop execution and entity locking, followed by query repair.

ReAct interleaves reasoning and actions (tool calls) for iterative evidence collection and correction [25]. Surveys on agentic RAG emphasize planning, memory, feedback, and bounded tool use to reduce drift [24, 26, 27]. MedHopQA’s emphasis on retrieval and cost-aware engineering, plus its short-entity output, makes a bounded agentic workflow suitable [13]. We adopt a ReAct-inspired design with explicit state and iteration caps.

### RAG-LLM evaluation methods

#### Classical QA metrics and semantic similarity metrics

EM and token-level F1 were popularized by SQuAD [28]. In biomedical QA, synonymy and naming variation motivate additional evaluation beyond strict string matching, reflected in BioASQ [6–8]. BERTScore uses contextual embeddings for semantic evaluation [29], but does not guarantee evidence support.

#### RAG-specific evaluation and faithfulness

RAG evaluation requires separating retrieval-side and generation-side failures and measuring groundedness (faithfulness) with respect to retrieved context. Surveys on RAG evaluation summarize metrics for retrieval relevance, context quality, answer correctness, and faithfulness, including LLM-as-a-judge approaches and their limitations [30, 31]. RAGAS proposes reference-free evaluation for RAG pipelines with metrics such as faithfulness and answer relevance computed using LLM-based judgements [32]. These evaluation frameworks motivate internal validation modules, but they also imply that ablation and error analysis are needed to attribute improvements to retrieval, reranking, or control logic rather than to judge behaviour alone.

#### MedHopQA evaluation metrics

MedHopQA evaluates short answers using two metrics: EM and a MedCPT-based concept-level score [13, 19]. Strict normalization applies: lowercasing, punctuation removal, synonym mapping. Concept-level score evaluates semantic equivalence of predicted and reference biomedical concepts.


(1) \begin{eqnarray*}
EM = \frac{1}{N}\mathop \sum \limits_{i = 1}^N 1\left[ {\widehat {{a_i}} = {a_i}} \right]
\end{eqnarray*}

where $\widehat {{a_i}}$ is the normalized system output and ${a_i}$ is the reference answer.


(2) \begin{eqnarray*}
\textit{Concept Level} = \frac{{\textit{correct concepts}}}{{\textit{total concepts}}}
\end{eqnarray*}

This dual metric setup creates an objective for systems: produce canonical short answers that match strict formatting constraints for EM while maintaining biomedical concept correctness for concept-level evaluation. We planned to evaluate under both metrics and to attribute improvements through ablation rather than relying on judge-based metrics alone.

We report test-set EM under official CodaBench evaluation and conduct a systematic ablation study on the development set to isolate contributions of individual components in the *Retrieval metrics on development set* section.

### RAG-LLM benchmarks in biomedical knowledge domain

BioASQ is a foundational shared task for biomedical semantic indexing and QA and provides expert questions and gold answers [6, 7]. The BioASQ-QA corpus description provides additional documentation of curated resources and evaluation requirements that reflect biomedical synonymy and normalization [8]. These benchmarks often draw evidence from biomedical literature and curated resources, which differs from Wikipedia-derived benchmarks in distribution and entity surface forms.

PubMedQA evaluates QA over PubMed abstracts with yes/no/maybe labels and targets reasoning about research text [9, 33]. This differs from entity-centric short-answer tasks and typically operates over constrained evidence.

MedQA is a large-scale dataset based on medical exams and is widely used to evaluate clinical reasoning and medical knowledge [34]. MedMCQA provides a large multi-subject medical entrance exam dataset in multiple-choice format [35]. These benchmarks test clinical knowledge under answer-option constraints and are structurally different from open-ended, entity-centric, retrieval-based QA.

HotpotQA, 2WikiMultiHopQA, and MuSiQue provide multi-hop reasoning benchmarks over Wikipedia and motivate decomposition, iterative retrieval, and agentic workflows for multi-document evidence integration [4, 5, 22]. Multi-hop retrieval methods such as beam retrieval make the retrieval process explicit as a chain construction problem rather than independent top-k selection [23]. These works inform system designs for biomedical multi-hop QA even when the biomedical benchmark uses a different corpus.

MedHopQA evaluates multi-hop biomedical QA using Wikipedia-derived question–answer pairs curated from two connected Wikipedia pages, requiring short answers of at most three words and using EM and MedCPT concept-level evaluation [13]. Because the benchmark is Wikipedia-grounded, Wikipedia-based retrieval corpora and retrieval-heavy system designs are a natural fit [13, 14]. Our initial system used hybrid retrieval with fusion and prompt decomposition [14]. The present work modifies that system by replacing prompt decomposition with an agentic workflow with bounded hops, reranking, query repair, and answer-type validation while keeping the same Wikipedia corpus (see the *Corpus selection and processing* and*Agentic system design and prompts; multi-hop agentic RAG pipeline*sections).

MedHopQA includes rare disease content [13]. Orphanet provides rare disease nomenclature and ORPHAcodes [36, 37]. We integrated Orphanet as a safety mechanism: a gated list of synonyms and gene symbols reduces the risk of fabricating incorrect gene names when Wikipedia context is thin. Its effect on aggregate test-set EM is negligible but prevents some high-severity errors on rare disease items (see the *Orphanet Impact on Rare Disease Questions* section). Benchmark families are summarized in Supplementary Table S1.

### Prior work

Biomedical multi-hop QA has been explored in knowledge graph settings, where systems traverse multi-relation paths [38], and as multi-stage pipelines over curated resources [39]. Recent generative QA systems increasingly use RAG and agentic tool-use to reduce hallucinations. For example, BioRAGent employs a multi-agent system (guide, retriever, reviewer) to query structured databases (HPO, NCBI) and return precise answers [40]. In contrast, MedHopper is designed for the MedHopQA task, which requires reasoning over unstructured Wikipedia text to synthesize short, entity-centric answers from multiple documents [13].

Our initial BioCreative IX submission (DecompRAG) used hybrid retrieval with reciprocal rank fusion and prompt decomposition, achieving EM = 0.43 on the test set [14]. Decomposition can propagate early errors, and short-answer tasks are sensitive to answer-type and generic outputs, issues not reliably prevented by decomposition prompts alone. MedHopper replaces decomposition with a bounded state machine workflow that interleaves retrieval, intermediate entity extraction, validation, and query repair.

Several individual techniques have precedents: LLM-based output evaluation [41], reasoning-type categorization [4], and question/answer-type classification [42]. However, MedHopper’s use differs fundamentally:

- Validation is an in-loop control signal, not an offline metric [41]. Failed validation triggers a bounded query-repair step that preserves the predicted answer type and strategy; closed-loop behaviour absent from post hoc evaluation.
- The four execution strategies are not descriptive labels [4] but executable modules that govern constraint extraction, hop planning, entity sanitisation, and entity locking, operationalizing strategy decisions end-to-end.
- Answer-type enforcement is continuous, fine-grained (chromosome, gene, protein, etc.), and feeds back into repair, unlike front-end classifiers that label only at input time [42].

Thus, the novelty of MedHopper lies not in any single component but in their integration into a bounded state machine that jointly manages retrieval, reasoning, answer-type conformity, and repair under strict exact-match constraints. We report test-set EM and ablation results, showing that capped multi-hop execution, reranking, and query repair provide the largest gains. Qualitative analysis identifies answer-type inconsistency and surface-form instability as persistent challenges. Orphanet is used as a conservative harm-prevention mechanism for rare disease questions.

## Materials and methods

### Corpus selection and processing

We used the same Wikipedia-derived biomedical corpus as in our initial MedHopQA submission [14]. The corpus was obtained from a Wikipedia articles dump (current revisions only, excluding talk, and user pages) and filtered to health-related pages using category membership. Category sets were collected with PETScan. The resulting subset contained on the order of 225 000 health-related pages (including stubs), as reported previously. We retained this corpus and preprocessing so that changes in performance can be attributed primarily to the inference-time pipeline modifications rather than to differences in knowledge source.

For retrieval, articles were split into fixed-length character chunks. We kept the same conservative configuration used earlier: chunk size 512 characters with 32-character overlap [14]. Each chunk was embedded using MedEmbed-small-v0.1 with embedding normalization enabled and indexed in a local Chroma vector store. Unlike the initial submission, which used a hybrid lexical + dense setup, the present system uses dense retrieval only.

### Agentic system design and prompts; multi-hop agentic RAG pipeline

**Figure 1:**
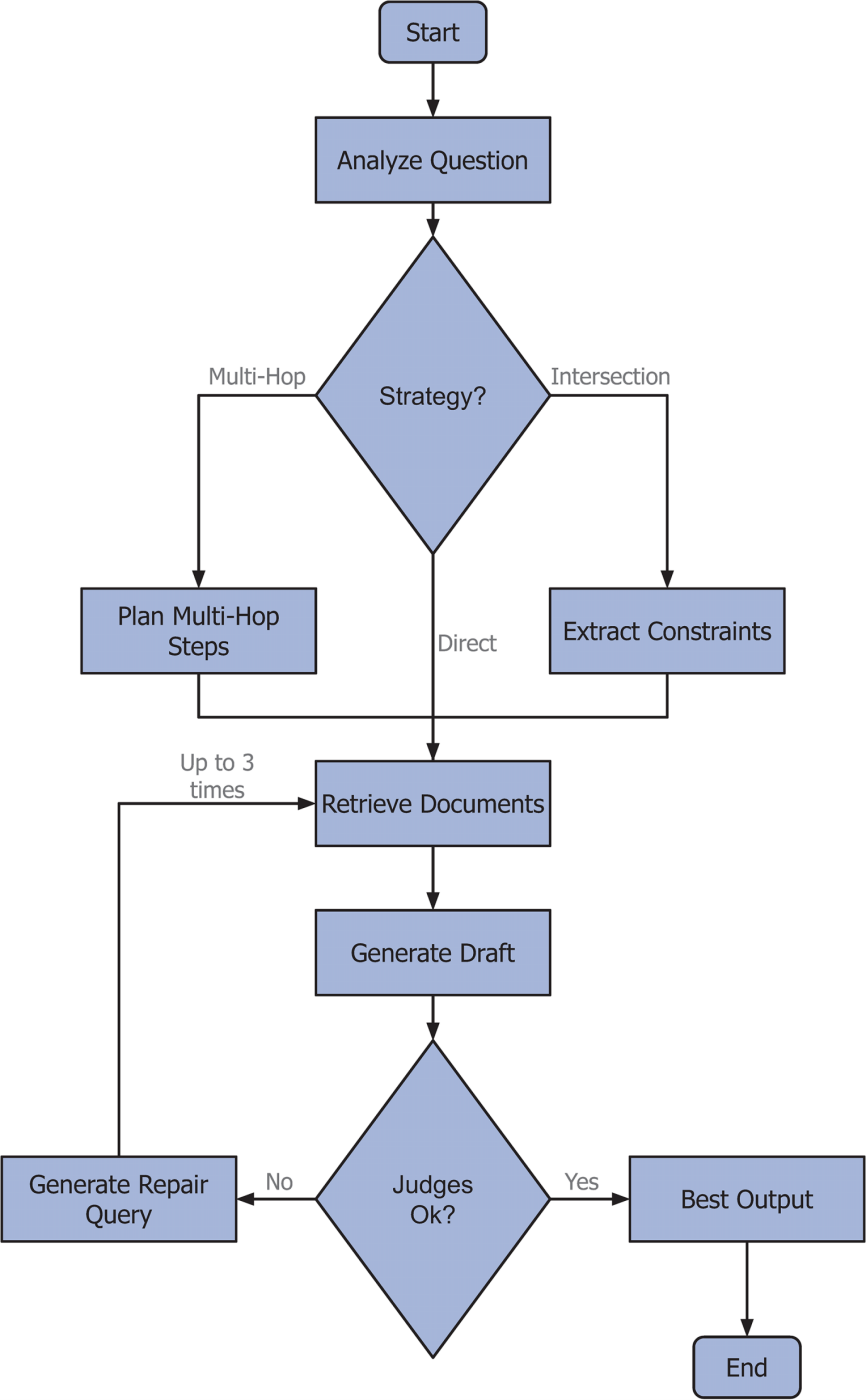
The system implements a bounded state machine with explicit termination criteria. Given a question, MedHopper: (i) routes it to one of four strategies (direct, definition, intersection, and multi-hop) and predicts answer type; (ii) constructs strategy-dependent queries; (iii) retrieves dense candidates (*k* = 90), merges across queries, reranks up to 200 candidates with FlashRank [21], and retains top 20 passages; (iv) generates either intermediate hop entities (capped at three hops, validated) or final answer; (v) applies multi-stage validation (self-reference, answer-type matching, grounding); and (vi) if validation fails, generates up to three repair queries conditioned on current state.

MedHopper is a bounded iterative workflow that replaces the hybrid retrieval and prompt decomposition of our earlier system [14]. It alternates between retrieval, intermediate entity extraction, answer generation, validation, and query repair, with explicit termination criteria to limit error propagation. The simplified workflow is illustrated in Fig. 1.

We do not present MedHopper as an unconstrained autonomous agent; it is closer to an agentic-style tool-using pipeline, where retrieval is treated as an external action inside a controlled loop. This design is aligned with the general idea in tool-using workflows such as ReAct (interleaving intermediate reasoning with actions), but our implementation is deliberately bounded and task-specific [25].

Retrieval is performed over the dense Chroma index (see the *Corpus selection and processing* section). For each retrieval call, we use cosine similarity selection with a relatively large candidate set (*k* = 90). Retrieved candidates from multiple query strings are merged to remove near-duplicates and then reranked with FlashRank [21]. To keep reranking tractable, at most 200 unique candidates are reranked per question, and the top 20 are retained as the evidence context for subsequent model calls.

Query construction is strategy-dependent:

- In direct and definition modes, retrieval is driven primarily by the question text (and, if generated later, an additional repair query).
- In intersection mode, the system first extracts short constraint phrases by asking the model to copy them verbatim from the question, then uses those phrases as additional retrieval queries; extracted phrases are filtered to ensure they occur in the original question text.
- In multi-hop mode, the system first asks the model to produce up to three hop subquestions, each intended to return a single short entity. Those hop lines are sanitized to remove duplicates, low-content output, or non-question text. The retrieval process alternates between two phases: gathering evidence to answer the next unresolved hop and, once all hops are complete, gathering evidence to support the final answer. When intermediate entities have been extracted, they are used to steer later retrieval by being concatenated into subsequent queries, but only after passing a conservative validation filter.

For each hop, the model is instructed to output a single specific entity (not a category) using only the evidence context. Intermediate hop answers are appended to a ‘locked entities’ list only if they appear specific enough: the code rejects empty outputs, yes/no tokens, generic category words, and obvious placeholders (‘unknown’, ‘n/a’, etc.). This validation is intended to avoid locking on non-entities or overly generic terms that would misdirect later retrieval. For hops explicitly typed as yes/no, the system forces the output to exactly ‘Yes’ or ‘No’ and does not lock it as an entity.

To support rare disease naming and gene linkage, MedHopper optionally integrates Orphanet resources as a controlled query-expansion and candidate-hint mechanism. Orphanet provides standardized rare disease terminology and identifiers (ORPHAcodes) via the nomenclature pack, together with product files that include curated disease–gene associations [36, 37]; the application of ORPHAcodes in rare disease coding has been discussed by Mazzucato *et al*. [37]. In the current pipeline, Orphanet is consulted only to generate auxiliary query terms and candidate gene symbols; its output is never treated as direct evidence. Expansion is gated by two criteria: (i) the anticipated answer type must belong to a restricted set (specified in Supplementary Table S2), and (ii) a lightweight LLM prompt explicitly assesses whether the question likely refers to a rare or orphan medical entity that would benefit from terminology expansion. To limit noise and preserve efficiency, expansion is further constrained by configurable caps on the number of matched disorders, genes per disorder, and total expansion terms; only a subset of these terms is injected into retrieval and reranking queries. Additionally, when a hop targets a gene, the system may inject a short, Orphanet-derived list of candidate gene symbols into the hop prompt, accompanied by an explicit instruction that candidates must be corroborated by the retrieved context before being accepted.

All answer-generation prompts enforce a strict short answer: the model must output only the short answer, match the requested answer type, and return a specific entity rather than a generic category. After drafting an answer, MedHopper runs a validation sequence that combines rule-based checks and LLM-based judgements. The system checks that the answer is not self-referential, that it matches the requested type (including a rule-based rejection of generic answers such as ‘gene’ or ‘press’), and that it is supported by the retrieved context. For a small set of answer types where format ambiguity is common (e.g. durations), the system additionally requires explicit support in context via either substring support or a judge prompt that checks for an exact supporting span. If validation fails, the system invokes a query repair step.

The repair generator receives the original question, the predicted answer type, the current strategy, the next unresolved hop question (if in multi-hop mode), and the most recent validated locked entity. It is prompted to produce a short retrieval query targeting a specific entity. Several answer-type-specific rules are enforced:

- for chromosome, the query must include the word ‘chromosome’;
- for press_or_publisher, it must contain ‘press’ or ‘published’ and ‘organization’;
- for medical_specialist, at least two of the terms diagnosis, management, treatment, specialist, physician, doctor are required;
- for procedure, two of diagnosis, diagnostic, test, procedure, confirmed, imaging are required.

When a locked entity is present, it must appear verbatim in the generated query. After generation, if the locked entity is missing, it is prepended, and any phrases that could redirect the search, such as ‘besides’, ‘other than’, or ‘instead of’, are removed. The resulting query is used to repeat retrieval and answer generation. The repair step does not alter the strategy or answer type; it only refines the retrieval query. The repair loop is capped at three attempts. The first candidate answer that passes all validation checks is stored as the best answer and returned even if later attempts do not succeed; if no answer is validated after three cycles, the system returns the best previously validated answer, or ‘null’ if none exists.

### Ablation study design

To isolate the impact of specific architectural choices and hyperparameters, we conducted an ablation study on the MedHopQA development set (*N* = 45). All configurations utilize the same corpus and index described in the *Corpus selection and processing* section and are evaluated using EM.

We verified stability across five random seeds {42–46} for all 14 configurations. Given our use of greedy decoding (temperature = 0) and fixed seeds for retrieval components, we hypothesized perfect reproducibility (Supplementary Table S3). Consequently, unless noted otherwise, we report test-set results (*N* = 1000) using a single canonical seed 42, since the end-to-end pipeline is deterministic under our decoding and retrieval settings. We therefore treat multi-seed test-set evaluation as unnecessary for this configuration and instead allocate budget to ablations and qualitative error analysis. The development set (*N* = 45) is used solely for controlled within-study comparisons across configurations; final performance claims are based on the official test set (*N* = 1000)

We compare the full pipeline against a Single-Pass baseline (no multi-hop planning or repair). Within the full pipeline, we ablate the Repair Loop (three steps vs 0) and four Validation Modules (generic filter, answer type validation, grounding check, and self-reference check) by disabling them individually. For Orphanet integration, we compare expansion enabled vs disabled, vary the expansion cap {5, 10, 20}, and disable gene hints (injection of candidate gene symbols into hop prompts). Finally, we vary reranking depth ${k_r}$ ∈ {10, 20, 30}, and disable the reranker entirely. The Full Pipeline baseline uses default settings (${k_r}$ = 20, repair enabled, all validators active).

We report results under two evaluation settings. First, for the MedHopQA test set (*N* = 1000), EM is computed using the official CodaBench contest evaluation, which applies the benchmark’s answer normalization and synonym handling. Second, for the development set used in our ablations (*N* = 45), we compute EM locally using an exact string match protocol without synonym lists (i.e. stricter than the official EM). Unless explicitly stated otherwise, all test-set EM values in the *Retrieval metrics on development set* section refer to the official CodaBench score, whereas seed sensitivity and ablation trends reported on the development set in the *Seed sensitivity and determinism analysis* section use the local strict EM. These two EM values are therefore not directly comparable in absolute terms, but the local setting is sufficient for controlled within-study comparisons across ablations because it uses a fixed evaluation script and identical inputs for all configurations.

## Results

All experiments were conducted on a standard desktop workstation without specialized server hardware. Hardware details are provided in Supplementary Table S4. We chose Qwen2.5-7B due to its strong performance on general and scientific reasoning benchmarks at the time of system development, and its permissive licence. The 7B parameter scale offered a balance between inference speed on consumer hardware (RTX 5080) and the reasoning capacity required for multi-hop tasks.

### Seed sensitivity and determinism analysis

The system exhibits perfect determinism (standard deviation of EM = 0.000, instance-level agreement 100%, pairwise Cohen’s κ = 1.0) across five random seeds on the development set. Full results of all ablation configurations on the development set are given in Supplementary Table S3.

### Retrieval metrics on development set

We computed recall@k (*k* = 1, 5, 10, 20) on the development set (*N* = 45). The highest recall@20 (0.778) is achieved with Rerank Top-N = 30 and with Orphanet Expansion Off. Detailed metrics for all retrieval-affecting ablations are presented in Supplementary Table S4. Because gold answers are not publicly available for the test set, all retrieval metrics are reported only on the development set.

### Ablation study results on MedHopQA contest

To evaluate the contribution of individual components within the MedHopper pipeline, we conducted a systematic ablation study on the MedHopQA test set (*N* = 1000). All configurations were submitted to CodaBench and evaluated using the official EM metric (seed 42). Table 1 reports EM scores for the full system and configurations with specific modules disabled or modified. The full pipeline achieved an EM score of 0.55. While individual components yielded modest gains in isolation, the results suggest that system performance depends on the coordinated interaction of iterative reasoning and retrieval validation, rather than on any single dominant mechanism.

**Table 1:** Ablation study results on MedHopQA test set (*N* = 1000).^a^

Ablation configuration	^EM score (*N* = 1000)^	Change in EM compared to full pipeline
Full Pipeline	0.55	–
Architecture		
Single Pass (no multi-hop)	0.50	−0.05
Repair Off	0.52	−0.03
Validation Modules		
No Generic Filter	0.54	−0.01
No Answer Type Validation	0.54	−0.01
No Grounding Validation	0.54	−0.01
No Self-Reference	0.54	−0.01
Orphanet Knowledge		
Orpha Expansion Off	0.54	−0.01
Orpha Gene Hints Off	0.54	−0.01
Orpha Expansion Cap 5	0.54	−0.01
Orpha Expansion Cap 20	0.54	−0.01
Retrieval and Reranking		
Rerank Off	0.52	−0.03
Rerank Top-N = 10	0.51	−0.04
Rerank Top-N = 30	0.54	−0.01

a All configurations evaluated using EM with seed 42. Full Pipeline uses Rerank Top-N = 20.

For context, Table 2 shows the official test phase leaderboard for EM. MedHopper achieves 55.0, remaining below the baseline and unchanged in rank relative to our prior DecompRAG system (43.9).

**Table 2:** Official test phase EM results on MedHopQA (CodaBench).^a^

Team name	EM score
DMIS Lab	87.3
UETQuintet	83.8
Insilicom	80.0
PreceptorAI	73.4
Fluxion	68.1
CLaC	67.6
Nckuiirlab	66.9
Baseline	59.1
**MedHopper (this work) **	**55.0**
Biojay	45.8
Orekhovichi (DecompRAG)	43.9
LasigeBioTM	28.3
LaosFun	26.1
BioHop	20.7
CaresAI	18.6

a Baseline is a zero-shot GPT-4o model with a straightforward prompt. Orekhovichi corresponds to our initial DecompRAG submission. MedHopper is the proposed system in this work, its name is given in the Table in bold.

### Routing strategy distribution

We recorded the strategy predicted by the routing module for each question in the test set (*N* = 1000) when running the full pipeline. Table 3 shows the distribution.

**Table 3:** Distribution of routing strategies on the MedHopQA test set.

Strategy	Count	Percentage
Direct	379	37.9%
Definition	352	35.2%
Intersection	13	1.3%
Multi-hop	256	25.6%

More than half of the questions are routed to direct or definition (73.1% combined), whereas only 25.6% trigger the full multi-hop loop. However, the routing prompt is deliberately short and contains no examples or explicit bias towards the multi-hop strategy; it may therefore be too conservative, labelling questions as direct or definition even when the intended MedHopQA construction involves two Wikipedia pages. Because the dataset does not provide ground-truth strategy labels, we cannot determine the true proportion of multi-hop questions. The observed distribution therefore reflects a combination of the dataset characteristics and the current routing design. The intersection strategy is rarely used (1.3 %), as expected for questions that explicitly mention multiple constraints on the same entity.

### Qualitative error example analysis results

To complement the quantitative ablation results, we conducted a qualitative analysis of system outputs across ablation configurations. This analysis aims to identify patterns of failure, understand the conditions under which individual components contribute most to performance, and characterize the types of errors that persist even in the full pipeline configuration.

**Figure 2:**
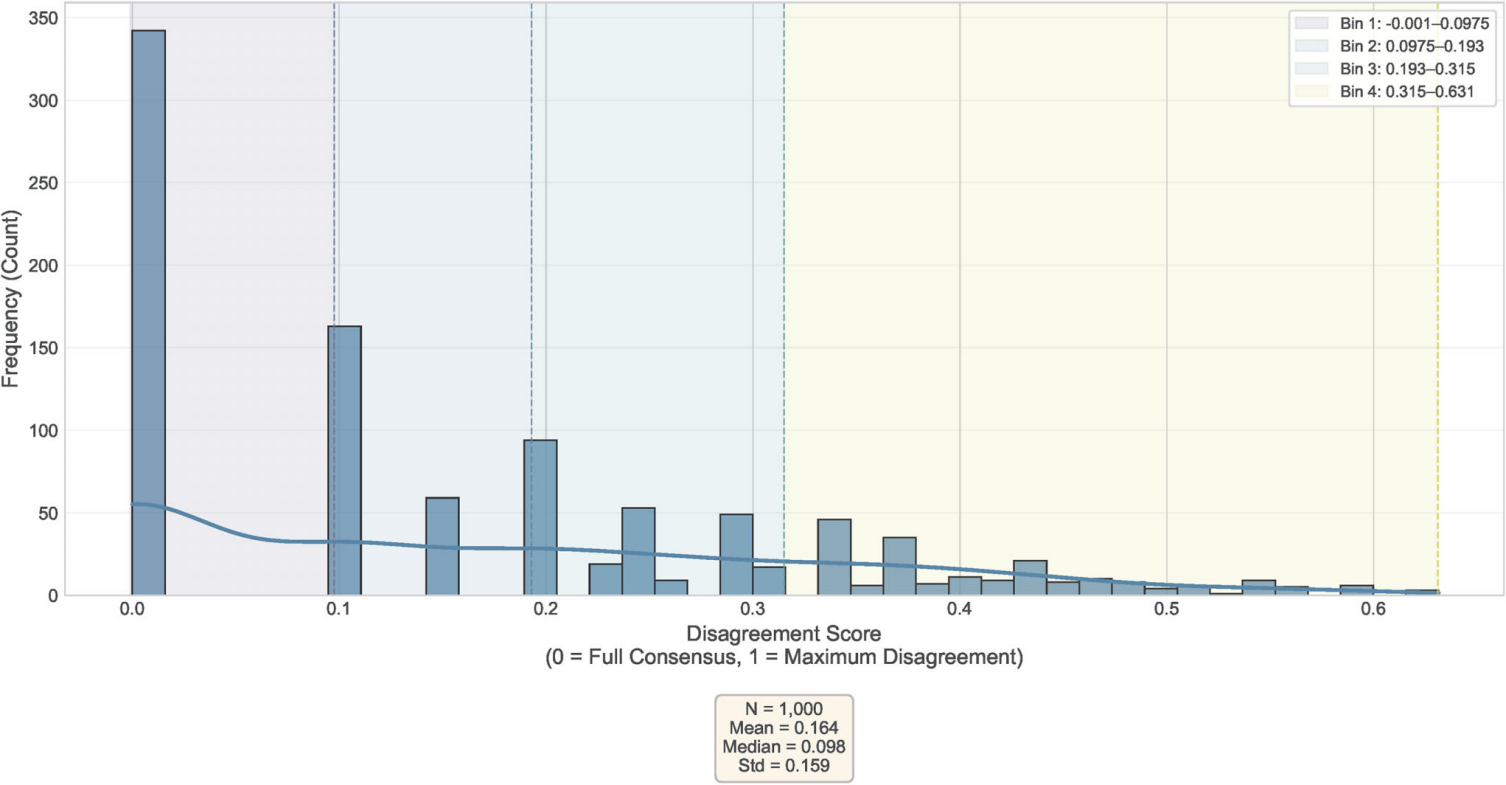
Distribution of disagreement scores; computation details are provided in Appendix A.1.

Rather than selecting questions arbitrarily or based solely on maximum disagreement, we employed a stratified sampling approach based on disagreement across ablation configurations. Specifically, we compute an entropy-based disagreement score over the set of answer strings produced by all ablations for each test-set question, and we partition questions into quantile-based strata of this score (Table 4). Within each stratum, we select representative questions near the stratum’s median disagreement to avoid over-representing either trivial consensus cases or extreme instability. Implementation details of the disagreement score, the density visualization shown in Fig. 2, and the representative item selection procedures are provided in SupplementaryAppendix A.1.

**Table 4.** Representative questions selected from each disagreement stratum.^a^

Stratum	Score range	*N* questions selection
Consensus (Bin 1)	$( { - 0.001,\;0.0975} ]$	25
Low–moderate (Bin 2)	$( {0.0975,\;0.193} ]$	25
Moderate (Bin 3)	$( {0.193,\;0.315} ]$	25
High–moderate (Bin 4)	$( {0.315,\;0.631} ]$	25

a The selection procedure is described in Supplementary Appendix A.1.

The disagreement score quantifies answer instability across ablation configurations using normalized Shannon entropy, and the stratified sampling procedure ensures balanced coverage of consensus and divergent cases.

### Orphanet impact on rare disease questions

To assess whether the Orphanet integration benefits the rare disease content of MedHopQA, we used the same 100 randomly sampled test-set questions that served as the basis for the disagreement analysis and Supplementary  Appendix A.7. We manually annotated each question for relevance to rare diseases. Of the 100 questions, 47 were classified as rare disease related. The full pipeline (Orphanet enabled, default cap 10, gene hints on) was compared against four Orphanet-ablated configurations: expansion disabled, expansion cap 5, expansion cap 20, and gene hints disabled. Table 5 reports EM on the rare disease subset.

**Table 5:** EM scores on rare disease questions (*N* = 47).^a^

Configuration	Correct	Accuracy
Full pipeline (Orphanet enabled, cap 10, hints on)	26	55.3%
Orpha Expansion Off	20	42.6%
Orpha Expansion Cap 5	20	42.6%
Orpha Expansion Cap 20	20	42.6%
Orpha Gene Hints Off	20	42.6%

a All ablated variants performed identically.

The full pipeline outperforms all Orphanet-ablated configurations by six correct answers, corresponding to a gain of 12.7 percentage points (55.3% vs 42.6%). The ablated variants are indistinguishable from each other, indicating that the presence of expansion (with the default cap) matters more than the exact cap value. This secondary analysis demonstrates that Orphanet integration may improve retrieval, even though its overall contribution to the full test set is diluted.

## Discussion

### Component contributions and interactions

The largest performance decrement (−0.05 EM) occurred when the multi-hop architecture was replaced with a single-pass retrieval approach. This finding supports the hypothesis that a substantial subset of MedHopQA questions requires sequential evidence gathering, wherein intermediate entities must be resolved before a final answer can be constructed. For example, questions asking for the chromosomal location of a gene implicated in a rare disease necessitate first identifying the relevant gene before retrieving its genomic coordinates. The single-pass baseline, which retrieves context and generates an answer in one step, lacks the capacity to decompose such dependencies, leading to higher error rates.

Disabling the query repair mechanism resulted in a −0.03 EM reduction. The repair loop is designed to reformulate retrieval queries when initial attempts fail to yield answers that satisfy validation criteria. Its modest but consistent contribution suggests that while many questions can be answered on the first retrieval attempt, a non-trivial fraction benefits from iterative refinement. One plausible explanation is that early retrieval failures often stem from lexical mismatches or ambiguous phrasing; the repair step, by incorporating previously identified entities and constraints, can steer subsequent searches towards more relevant passages. However, the limited magnitude of improvement also indicates that repair effectiveness is bounded, potentially by the quality of the initial question analysis or by inherent limitations in the retrieval corpus.

Ablating individual validation modules (generic answer filtering, answer-type validation, grounding checks, and self-reference detection) each reduced EM by ∼0.01 in isolation. These effects are small but consistent, suggesting that the validation stage acts as a conservative gate that helps prevent a subset of clearly invalid outputs (e.g. generic category words, type/format mismatches, or answers not supported by the retrieved context). However, because we ablate each validator independently, these results do not establish whether the validators address disjoint error modes or substantially overlap; interaction effects would require a targeted factorial analysis. A practical implication is that validation can improve strict EM by preventing catastrophic format/type errors, but may also reject partially correct answers under heuristic thresholds (see the *Error modes under exact-match scoring*section).

The insensitivity of results to expansion caps (5 vs 20 terms) may suggest a saturation point wherein additional terminology neither harms nor helps retrieval quality. This may imply that the primary bottleneck for these questions lies not in lexical coverage but in the system’s ability to reason over retrieved evidence.

Retrieval configuration exhibited greater sensitivity than domain expansion. Disabling cross-encoder reranking led to a −0.03 EM drop, indicating that while initial retrieval captures a broad set of potentially relevant passages, reranking is critical for surfacing the specific snippets required for exact-match answers. Varying the number of documents passed to the answer generator revealed a performance optimum at 20 documents (Full Pipeline): reducing this to 10 decreased EM by −0.04, likely truncating necessary context for multi-hop reasoning, whereas increasing to 30 provided no benefit. A possible explanation is that beyond 20 documents, additional context does not improve evidence specificity and can interfere with short-answer extraction. Many remaining errors are not ‘wrong topic’ errors but answer-type errors (e.g. chromosome vs cytoband, process vs procedure, yes/no vs symptom), which are heavily penalized under exact string matching.

### Error modes under exact-match scoring

On this slice, the component effects are not subtle; a small number of questions account for a large share of the qualitative damage because the ablations push the output into a different answer type or an idiosyncratic surface form that is unlikely to coincide with a hidden EM vocabulary.

#### Observed patterns

Several Bin 4 items show consistent chromosome/cytoband confusion. QIDX 136660 asks for the chromosome of a gene involved in a rare overgrowth syndrome. The full pipeline returns Chromosome 11 (incorrect). Many ablations produce 11p15.5 or Chromosome 11p15.5 (also incorrect), and one configuration outputs the gene symbol GPC3 (incorrect). No configuration yields the correct answer for this question. This pattern repeats for other ‘on which chromosome’ questions. QIDX 926927 asks for the chromosome of a ryanodine receptor gene. The full pipeline gives Chromosome 19 (incorrect), while some ablations return Chromosome 1q42.1-q43 (this answer is correct; for example, rerank_topn_10 and rerank_topn_30 achieve exact match). Other settings drift to Chromosome 14 or Chromosome 19q132 (both incorrect). The system thus alternates between multiple locus representations (chromosome-level vs cytoband-level), and only certain ablations recover the correct genomic coordinate.

A second recurring failure mode is answer-type inconsistency on yes/no and definition-style prompts. QIDX 520077 (yes/no) is stable as Yes in the full pipeline (this is correct), but multiple ablations output Headaches or Glare, which are topically related but structurally incompatible, yielding maximal EM errors. QIDX 854124 asks for the essential process of forming new blood cellular components. The full pipeline answers Leukopoiesis (incorrect). Many ablations give Haematopoiesis (this is the correct answer; for example, no_generic_filter and no_grounding_validation achieve it), while others jump to Phagocytosis or Bone marrow transplantation (incorrect). The correct answer is a near-synonym of the full pipeline’s output, but the latter is not accepted under EM scoring.

The data also contain examples where ablations change not only form but the underlying hypothesis class. QIDX 331013 (protein whose deficiency increases aneurysm risk) yields MMP16 in the full pipeline (incorrect). All ablations produce TGFbeta1, ELANE, or Neutrophil elastase (none are correct). Similarly, QIDX 643490 (LGMD subtypes with decreased dystrophin) gives FKTN in the full pipeline (incorrect), and ablations vary across LGMD2D, LGMD2C, and LGMD2I (all incorrect). These are different candidate entities rather than alternate spellings.

There are cases where the full pipeline itself is misaligned to the requested entity type, and ablations expose that misalignment without correcting it. QIDX 280426 asks for an immune marker on chromosome 6 used to diagnose juvenile idiopathic arthritis. The full pipeline answers Chromosome 6 (a location, not a marker; incorrect). Other configurations propose CD69 or HLA-B (marker-like strings), but none are correct according to the gold answer.

Conversely, the low-disagreement bin (Bin 1) behaves as expected: many questions are invariant across ablations and correctly answered. QIDX 190359 always yields Acoramidis (correct), QIDX 142159 always yields NEB (correct), and QIDX 657453 always yields cutaneous T-cell lymphoma (correct). These items indicate that when the retrieved corpus contains a single prominent lexical form for the gold answer, the pipeline configuration matters little. Instability concentrates in higher-disagreement items, where retrieved evidence contains multiple plausible strings (synonyms, abbreviations, loci at different granularity, related entities), and the components primarily govern which string is selected as the short answer. However, correctness is not guaranteed, as many of those selections are still wrong.

#### Interpretation

Taken together, the ablation behaviour on these specific questions suggests that the components are primarily shaping: (i) whether the output stays within the requested answer type (chromosome vs cytoband vs gene; process vs procedure; yes/no vs symptom) and (ii) which surface form is emitted when multiple plausible lexicalizations are present. The representation drift observed in chromosome/cytoband confusion persists even when retrieved evidence is locally consistent. For questions where the retrieved corpus contains multiple adjacent plausible answers, the configuration choices essentially determine which specific string the system selects as its final output.

### Limitations

Despite the modular design, the full pipeline’s strict EM score of 0.55 indicates that a substantial fraction of benchmark questions remain unsolved under this evaluation setting. Across ablations, the largest effects are associated with the multi-hop execution loop, reranking, and query repair, whereas validation and controlled expansion contribute smaller, context-dependent changes. Several limitations warrant caution in interpretation. First, the validation logic may discard partially correct answers that fail a single heuristic, potentially underestimating the system’s factual coverage. Second, the repair mechanism relies on the language model to diagnose retrieval failures; if the model misattributes the cause of failure, subsequent queries may drift further from the target. Third, the category-matching approach used for corpus construction may have inadvertently filtered out evidence essential for solving certain benchmark items.

In summary, the ablation study underscores that agentic, multi-hop reasoning provides a measurable advantage for complex medical question answering, but that gains from individual components are context-dependent and often modest. Future iterations might explore soft validation strategies, adaptive retrieval depths, or ensemble methods to better balance precision and recall. For now, these results suggest that added system complexity should be justified by clear improvements in robustness, not small gains in aggregate metrics.

### Retrieval metrics and multi-hop considerations

Supplementary Table S3 shows that on the development set (*N* = 45), disabling the reranker does not reduce recall@20 (0.756 in both Full Pipeline and Rerank Off). For these 45 questions, the relevant passage was already present in the top 20 without reranking. However, disabling reranking increases recall@1 from 0.267 to 0.356, meaning the correct passage becomes more often the first result. On the test set (*N* = 1000), reranking improves EM from 0.52 to 0.55 (Table 1). Two factors explain this apparent discrepancy.

First, recall@k only checks whether the gold answer string appears verbatim in a retrieved passage. MedHopQA questions require multi-hop synthesis: the correct answer may be spread across two Wikipedia pages, and even when the answer string is present somewhere, the LLM must still correctly identify and combine intermediate entities. The test-set EM gain captures this synthesis ability, whereas recall@20 does not.

Second, retrieval metrics cannot be computed on the test set because gold answers are not released. The development set (*N* = 45) is too small to detect small recall differences. Therefore, the absence of a recall@20 improvement does not contradict the test-set EM benefit. The reranker’s value likely lies in improving ranking quality (higher recall@1) and answer extraction specificity, not in expanding the total set of relevant passages. The test-set EM results remain the primary evidence for the reranker’s contribution.

## Conclusion

On the MedHopQA test set, MedHopper achieves an EM score of 0.55 under official CodaBench evaluation, improving from 0.43 in our initial submission. Ablation results indicate that performance gains are not attributable to any single dominant component but emerge from the coordinated interaction of several mechanisms. The largest contributions come from multi-hop execution, reranking, and the bounded query-repair loop. Individual validation modules provide smaller, consistent improvements, primarily by preventing answer-type errors such as returning a generic category when a specific entity is required. Reranking proves critical for surfacing the specific passages needed for exact-match answers, with performance degrading when it is disabled or when the context window is truncated. These gains are achieved while running entirely on consumer hardware, without cloud APIs or recurring costs.

Error analysis reveals that remaining failures under exact-match scoring are dominated by two related phenomena: surface-form instability and answer-type inconsistency. When retrieved evidence contains multiple plausible lexicalizations for the same biomedical concept (synonyms, abbreviations, representations at different granularity), the system’s component configuration can determine which specific string is selected. Similarly, disabling certain checks can cause the output to shift from the requested answer type (e.g. chromosome vs cytoband, process vs procedure, yes/no vs symptom) to a topically related but structurally incompatible form. These patterns suggest that the primary function of the workflow components is to constrain which string is emitted when multiple plausible candidates are present in the retrieved context, rather than to recover facts that are entirely absent from the corpus.

The novelty of MedHopper lies not in being the first to apply agentic RAG to multi-hop QA, but in its bounded implementation adapted to the strict constraints of the MedHopQA task: short, entity-centric answers evaluated by EM.

Several design constraints limit current performance. The system handles entity normalization only implicitly through retrieval scoring rather than explicit biomedical concept mapping, leaving synonym variation as a persistent error source. The fixed iteration budget and predefined strategy set cannot adapt to questions that fall outside the four routing patterns or require more than three hops. Validation modules rely on LLM-based judgements, introducing dependence on prompt stability and increasing inference cost without guaranteed gains under strict EM evaluation. Finally, because EM does not distinguish between semantically equivalent entities with different canonical surface forms, some observed errors may reflect normalization mismatch rather than factual misunderstanding.

Future work could address terminology variation by integrating entity linking based on Unified Medical Language System (UMLS) [43], a comprehensive biomedical thesaurus that maps synonyms to standardized concepts during query construction and answer postprocessing, improving robustness to synonym-rich questions without relaxing output constraints. Adaptive prompt strategies, such as conditioning few-shot examples on predicted question type, could reduce hop-generation errors. Corpus expansion would address errors stemming from missing knowledge.

## Supplementary Material

baag047_Supplemental_File

## Data Availability

The code for this study is available at https://github.com/RustT883/MedHopper.
