# Supplementary material for: MedHopper: an agentic RAG-LLM system for multi-hop biomedical QA
Source: Database (Oxford). 2026 Aug 13;2026:baag047. doi: 10.1093/database/baag047 (PMC13472165; doi:10.1093/database/baag047)
Supplement: Supplementary file 1 [file baag047_supplemental_file.docx]

**Supplementary Data**

**Appendix A.1. Representative Question Selection Process**

Rather than selecting questions arbitrarily or based solely on maximum disagreement, we employed a stratified sampling approach based on the normalized entropy disagreement score. This ensures our qualitative analysis represents the full spectrum of system behavior, from high-consensus to high-disagreement cases.

For each question $q_{i}$ in the test set, we computed a disagreement score $D(q_{i})$ based on the normalized Shannon entropy of answers across all ablation configurations:

$D(q_{i}) = \frac{H(A_{i})}{{log}_{2}(N_{i})}= \frac{-\sum_{j=1}^{k} p_{ij}{log}_{2}(p_{ij})}{{log}_{2}(N_{i})}$	(A1.1)

where: $A_{i}$ is the set of answers produced by all ablation configurations for question $q_{i}$; $N_{i}$ is the number of non-empty answers for question $q_{i}$; $k$ is the number of unique answer strings and $p_{ij}$ is the proportion of configurations producing answer $j$.

This score ranges from 0 (perfect agreement across all configurations) to 1 (maximum disagreement, where each configuration produces a unique answer). We normalized the results by ${log}_{2}(N_{i})$ to account for varying numbers of valid responses across all questions.

To characterize the global distribution of these scores, we estimated the probability density function using Kernel Density Estimation (KDE) with a Gaussian kernel:

$\hat{f}(x) =\frac{1}{nh}\sum_{i=1}^{n} K(\frac{x - D(q_{i})}{h})$	(A1.2)

where $K(\cdot)$ is the standard Gaussian kernel, $h$ is the bandwidth selected via Silverman’s rule, and $n$ is the number of questions. The resulting density estimate, overlaid on a histogram of observed scores, provides a smooth visualization of the disagreement scores (Figure 2).

Rather than defining bins using fixed thresholds, we partitioned the empirical disagreement distribution into quantile-based strata (Table 4).

For each stratum $S_{k}$, we then selected a balanced number of representative questions to avoid over-representing either trivial consensus cases or extreme instability. Within each stratum, we chose questions whose disagreement scores lie closest to the stratum’s empirical center (within-stratum median), with small random jitter used only to break ties:

${q^{*}}_{k}= arg\min_{q_{i}\in S_{k}}|D(q_{i})-median(D(q)): q\in S_{k}|$	(A1.3)

**Appendix A.2. Benchmark families in Biomedical QA Context**

Table S1 illustrates why MedHopQA presents distinct challenges. Unlike clinical exam benchmarks (MedQA, MedMCQA) that provide answer options, MedHopQA requires open-ended entity generation. Unlike abstract-based QA (PubMedQA), it demands evidence synthesis across multiple Wikipedia passages. And unlike general-domain multi-hop benchmarks (HotpotQA, 2WikiMultiHopQA), it must handle biomedical terminology variation under exact-match scoring.

Table S1. Summary of medical and multi-hop question answering datasets, organized by family, with details on evidence source, output format, size, and key characteristics

| Family | Benchmark | Evidence Source | Output Format | Reported Size | Key Characteristics |
| --- | --- | --- | --- | --- | --- |
| Expert-Curated | BioASQ (11–13) | Literature/ontologies | Exact answers | Large-scale tasks | Synonymy focus; not wikipedia |
| Abstract-Based | PubMedQA (35) | PubMed abstracts | Yes/no/maybe | 1k expert labeled, 61.2k unlabeled, 211.3k artificially generated QA instances | Single-abstract reasoning |
| Clinical Exam | MedQA (36) | Medical exams | Open-domain | ~12k, ~34k, ~14k questions in English, simplified Chinese and traditional Chinese languages | Medical reasoning and diagnosis |
| Clinical Exam | MedMCQA (37) | Medical exams, entrance exams | Multiple-choice | ~194k | Option-constrained recall |
| Multi-Hop | HotpotQA (4) | Wikipedia | Explainable short fragment | ~90k | General decomposition |
| Multi-Hop | 2WikiMultiHopQA (5) | Wikipedia | Short answers | ~192k | Iterative retrieval fit |
| Multi-Hop | MuSiQue (25) | Wikipedia | Short answers | ~113k | Agentic workflows |

**Appendix A.3. Full Answer Inventory**

Before retrieval, the system runs a routing step that assigns each question to one of four strategies: direct, definition, intersection, or multihop. Separately, the system also predicts an answer type from a fixed inventory, listed in Table S2. This answer type is used throughout the run to constrain prompts and postprocessing, because MedHopQA requires a short answer and is sensitive to type and formatting errors (6).

Table S2. Fixed inventory of answer types used for validation and postprocessing.

| Category | Description |
| --- | --- |
| chromosome | e.g., Chromosome 11 |
| gene | e.g., BRCA1 |
| gene_mutation | e.g., V600E |
| protein | e.g., p53, DNA polymerase |
| disease_or_syndrome | e.g., Marfan syndrome |
| symptom | e.g., fatigue |
| medication | e.g., ibuprofen |
| chemical | e.g., acetate |
| procedure | e.g., lumbar puncture |
| medical_specialist | e.g., neurologist |
| organism | e.g., Bordetella pertussis |
| anatomical_structure | e.g., mandible |
| field_of_biology | e.g., mycology |
| press_or_publisher | e.g., Oxford University Press |
| person | e.g., Richard Bright |
| duration | e.g., 2 days |
| yes_no | Yes or No |
| other | Catch-all for less common types |

**Appendix A.4. Detailed Results of Seed Sensitivity and Determinism Analysis**

Table S3. Seed Sensitivity Analysis on MedHopQA Development Dataset. The system exhibits perfect determinism: the standard deviation (σ) of EM scores is 0.000, instance-level agreement is 100%, and pairwise Cohen’s Kappa is 1.0 for every configuration

| Ablation Configuration | Mean EM  (N = 45) | Change in EM compared to Full Pipeline |
| --- | --- | --- |
| Full Pipeline | 0.556 | – |
| Architecture | |  |
| Single Pass (no multi-hop) | 0.467 | -0.089 |
| Repair Off | 0.533 | -0.023 |
| Validation Modules | |  |
| No Generic Filter | 0.556 | 0.000 |
| No Answer Type Validation | 0.556 | 0.000 |
| No Grounding Validation | 0.533 | -0.023 |
| No Self-Reference | 0.556 | 0.000 |
| Orphanet Knowledge | |  |
| Orpha Expansion Off | 0.556 | 0.000 |
| Orpha Gene Hints Off | 0.556 | 0.000 |
| Orpha Expansion Cap 5 | 0.556 | 0.000 |
| Orpha Expansion Cap 20 | 0.556 | 0.000 |
| Retrieval and Reranking | |  |
| Rerank Off | 0.489 | -0.067 |
| Rerank Top-N = 10 | 0.511 | -0.045 |
| Rerank Top-N = 30 | 0.533 | -0.023 |

The most significant performance drops occurred when removing the multi-hop architecture (Single Pass: −8.9%) or disabling the reranker (Rerank Off: −6.7%).

**Appendix A.5. Hardware Details**

Table S4. Hardware and performance specifications of the MedHopper system on consumer-grade hardware. All measurements reflect end-to-end inference using the full_pipeline configuration.

| Component | Specification / Performance |
| --- | --- |
| GPU | Nvidia GeForce RTX 5080 (16 GB VRAM) |
| Driver / CUDA | Driver 560.35.03, CUDA 12.6 |
| System Memory | 32 GB RAM |
| CPU | 11th Gen Intel i7-11700 (8 cores and 16 threads, 32 GB) |
| VRAM USAGE | < 12 GB (peak during inference) |
| Inference Latency | ~15-20 seconds per query (end-to-end, three hops) |
| LLM | Qwen2.5-7B (temperature = 0, sampling disabled) |

**Appendix A.6. Detalization of Retrieval Metrics on Development Set**

To assess evidence coverage, we computed recall@k (k = 1,5,10,20) and the proportion of questions where the gold answer appears in any retrieved passage. The pipeline is deterministic (temperature = 0, fixed seeds), so values are identical across five seeds; 95 % confidence intervals equal the point estimates. Table S5 reports these metrics for ablations that modify retrieval behaviour.

Table S5. Retrieval metrics on the MedHopQA development set (N = 45) for ablations affecting retrieval. Means across five seeds; standard deviation is zero for all configurations.

| Ablation Configuration | Recall@1 | Recall@5 | Recall@10 | Recall@20 |
| --- | --- | --- | --- | --- |
| Full Pipeline | 0.267 | 0.556 | 0.644 | 0.756 |
| Rerank Off | 0.356 | 0.644 | 0.711 | 0.756 |
| Rerank Top-N = 10 | 0.244 | 0.533 | 0.622 | 0.622 |
| Rerank Top-N = 30 | 0.244 | 0.533 | 0.644 | 0.778 |
| Single Pass (no multi-hop) | 0.244 | 0.533 | 0.600 | 0.756 |
| Repair Off | 0.244 | 0.533 | 0.600 | 0.733 |
| Orpha Expansion Off | 0.267 | 0.622 | 0.711 | 0.778 |

The highest recall@20 (0.778) is achieved with Rerank Top‑N = 30 and with Orpha Expansion Off. The lowest recall@20 (0.622) occurs when only the top‑10 passages are kept (Rerank Top‑N = 10). Disabling reranking does not change recall@20 (0.756 in both Full Pipeline and Rerank Off) but increases recall@1 from 0.267 to 0.356. The repair loop slightly reduces recall@20 from 0.756 to 0.733. The single‑pass baseline achieves the same recall@20 as the full pipeline (0.756), but lower recall@1 and recall@5. These numbers are descriptive; interpretation is deferred to Section 4.4.

Computing retrieval metrics on the test set is not possible because gold answers are not publicly available; the test set is evaluated only through the CodaBench platform. All retrieval metrics are therefore reported on the development set (N = 45). Furthermore, recall@k measures only whether the gold answer string appears as a substring in a retrieved passage. This is a necessary condition for multi‑hop QA but not sufficient, because questions often require synthesising information from two separate Wikipedia pages. The presence of the answer string in a passage does not guarantee that the LLM can correctly combine intermediate entities across multiple hops. Hence, the retrieval metrics in Table 4 should be interpreted as a lower‑bound estimate of evidence coverage, not as a direct predictor of end‑to‑end EM.

**Appendix A.7. Extended Analysis of Disagreement Bins and Per-Ablation Correctness**

We extend the analysis by linking disagreement to factual correctness. Tables S6–S9 report the distribution of routing strategies, per-bin accuracy for all ablations, full-pipeline accuracy by strategy, and the proportion of questions where all (or a majority of) ablations are wrong.

Table S6. Strategy distribution (%) per disagreement bin.

| Bin | Definition | Direct | Intersection |
| --- | --- | --- | --- |
| Bin 1: -0.001–0.0975 | 28.0 | 48.0 | 0.0 |
| Bin 2: 0.0975–0.193 | 32.0 | 32.0 | 4.0 |
| Bin 3: 0.193–0.315 | 56.0 | 20.0 | 0.0 |
| Bin 4: 0.315–0.631 | 16.0 | 52.0 | 0.0 |

Table S7. Accuracy (%) per bin for each ablation configuration.

| Ablation | Bin 1 | Bin 2 | Bin 3 | Bin 4 |
| --- | --- | --- | --- | --- |
| Full Pipeline | 80.0 | 60.0 | 36.0 | 16.0 |
| Architecture | | | | |
| Single Pass (no multi-hop) | 80.0 | 36.0 | 28.0 | 8.0 |
| Repair Off | 80.0 | 56.0 | 36.0 | 8.0 |
| Validation Modules | | | | |
| No Generic Filter | 80.0 | 48.0 | 40.0 | 20.0 |
| No Answer Type Validation | 80.0 | 48.0 | 36.0 | 12.0 |
| No Grounding Validation | 80.0 | 56.0 | 44.0 | 16.0 |
| No Self-Reference | 80.0 | 48.0 | 40.0 | 16.0 |
| Orphanet Knowledge | | | | |
| Orpha Expansion Off | 80.0 | 44.0 | 36.0 | 20.0 |
| Orpha Gene Hints Off | 80.0 | 48.0 | 40.0 | 16.0 |
| Orpha Expansion Cap 5 | 80.0 | 48.0 | 40.0 | 16.0 |
| Orpha Expansion Cap 20 | 80.0 | 48.0 | 40.0 | 16.0 |
| Retrieval and Reranking | | | | |
| Rerank Off | 80.0 | 44.0 | 24.0 | 28.0 |
| Rerank Top-N = 10 | 80.0 | 40.0 | 28.0 | 12.0 |
| Rerank Top-N = 30 | 80.0 | 56.0 | 36.0 | 16.0 |

Table S8. Full Pipeline accuracy by routing strategy

| Strategy | Correct / Total | Accuracy (%) |
| --- | --- | --- |
| definition | 15/33 | 45.5 |
| direct | 19/38 | 50.0 |
| intersection | 1/1 | 100.0 |
| multihop | 13/28 | 46.4 |

Table S9. Percentage of questions where all ablations are wrong, and where a majority (≤ 75%) are wrong, per bin.

| Bin | All ablations wrong (%) | Majority (≥75 %) wrong (%) |
| --- | --- | --- |
| Bin 1: -0.001–0.0975 | 20.0 | 20.0 |
| Bin 2: 0.0975–0.193 | 8.0 | 52.0 |
| Bin 3: 0.193–0.315 | 28.0 | 60.0 |
| Bin 4: 0.315–0.631 | 48.0 | 76.0 |

The disagreement score correlates strongly with factual correctness. Accuracy of the full pipeline drops from 80 % in Bin 1 to 16 % in Bin 4, and in Bin 4 nearly half of the questions (48 %) are answered incorrectly by every ablation configuration. High disagreement therefore signals not only surface instability (e.g., “Chromosome 11” vs “11p15.5”) but also a propensity to converge on wrong answers. Notably, disabling the reranker (rerank_off) raises accuracy in Bin 4 from 16 % to 28 %, indicating that cross‑encoder reranking may be beneficial for the most difficult questions. The single‑pass baseline performs worst overall, especially in Bin 4 (8 % accuracy). These results refine the qualitative observations in Section 4.2 by quantifying how often answer variation coincides with factual error.
